# Mesh removal and ventral hernia repair with long-term absorbable mesh in case of mesh infection

**DOI:** 10.1007/s10029-025-03475-9

**Published:** 2025-10-14

**Authors:** Gaëtan-Romain Joliat, Guillaume Passot, Benoit Romain

**Affiliations:** 1 Department of Visceral Surgery, Lausanne University Hospital CHUV, University of Lausanne (UNIL), Lausanne, Switzerland; 2 Department of Digestive Surgery, Hôpital de Hautepierre, Strasbourg, France; 3 Department of General Surgery and Surgical Oncology, Centre Hospitalier Universitaire Lyon Sud, 165, chemin du Grand Revoyet, Pierre-Bénite, 69495 France

**Keywords:** Infection, Abdominal wall surgery, Complications, Morbidity

## Abstract

**Purpose:**

Mesh infection after ventral hernia repair remains rare, but often necessitates mesh removal. This study evaluated the short- and long-term outcomes of a strategy involving explantation of the infected mesh and insertion of a long-term absorbable mesh in acute (AMI) and chronic mesh infections (CMI).

**Methods:**

A retrospective study was performed in two tertiary centers (01.01.2017–31.12.2024). All consecutive patients with ventral mesh infection who underwent mesh explantation followed by placement of a long-term absorbable poly-4-hydroxybutyrate mesh were included. Primary endpoint was hernia recurrence rate. AMI was defined as mesh infection within 90 postoperative days.

**Results:**

Twenty-nine patients were included: 14 AMI/15 CMI. Meshes were intraperitoneal in 9/14 (64%) AMI and in 13/15 (87%) CMI patients. Twelve (86%) and 13 patients (87%) had a new mesh implanted in the retromuscular space in the AMI and CMI groups, respectively. Four (28%) and 3 patients (21%) needed posterior component separations in the AMI and CMI groups. Median hospital stays were 10 (5–15) and 7 days (5–13) in both groups. Nine patients in each group (64%/60%) developed postoperative complications. Hernia recurrence rates were 3/14 (21%) and 0 in the AMI and CMI groups (mean follow-up 17 months). One mesh had to be explanted in the AMI group (0 in the CMI group).

**Conclusions:**

The presented strategy was associated with high short-term morbidity, but good long-term results, with low mesh explantation and hernia recurrence rates. A longer follow-up and inclusion of more patients is required to assess the risk of hernia recurrence and reinfection with greater hindsight.

## Introduction

Ventral hernia repair still induces a significant short- and long-term morbidity [1, 2]. One of the most feared complications after this operation is the infection of the mesh that often needs to be removed [3, 4]. Mesh infections after ventral hernia repair occur in 1 to 8% of patients [5, 6]. Mesh infections are more frequent after open repair (7–8%) [7, 8] than after minimally-invasive surgery (1–2%) [9, 10]. Several risk factors for mesh infection after hernia repair such as uncontrolled diabetes mellitus, obesity, active smoking, immunosuppressive medication including steroids, onlay (subcutaneous) mesh position, intraoperative bowel lesions, or postoperative surgical-site infections have been described [11, 12]. A postoperative surgical-site infection can contaminate the mesh depending on the location of the mesh (intraperitoneal, preperitoneal, retromuscular, or subcutaneous) and the depth of the infection [11]. After contamination, acute or chronic mesh infection can develop. Mesh infection is often polymicrobial, the most frequent pathogens being Staphylococcus aureus, Enterobacteriaceae, and anaerobic bacteria [13]. Once infection of the mesh occurs, it is often necessary to remove the mesh to definitely treat the infection [14]. Possibility of mesh salvage depends on various factors such as the type of involved bacteria, the timing of the mesh infection, and the type (long-term absorbable, synthetic or biological, pore size, material) and location of mesh that was used. In particular, polytetrafluoroethylene (PTFE) meshes have been shown to be more at risk of requiring explantation [11, 15]. Even though risk factors have been identified, the management of mesh infection currently remains controversial with no clear guidelines [5]. Some studies have shown that a large percentage of macroporous polypropylene infected meshes can be salvaged (up to 80%) when negative pressure wound therapy is used [4, 16], especially when located extraperitoneally, while other studies recommend mesh explantation for other mesh types or intraperitoneal location [17].

Recently, the use of long-term absorbable mesh has been shown to be potentially more resistant to infection than synthetic meshes, and to be safe when use in certain contaminated fields [18, 19].

For a couple of years now, in case of abdominal wall mesh removal due to infection, our management strategy was to use a long-term absorbable mesh to reinforce the abdominal wall. The long-term absorbable mesh is either put at the same time as mesh removal (single-stage management) or after a couple of days following subcutaneous vacuum-assisted (VAC) therapy during a second operation (two-stage procedure) [19, 20]. Using this strategy, it was hypothesized that antibiotherapy could only be for a short time window (therefore potentially decreasing the inherent risk of secondary effects and allergies), patients could have shorter length of stay (LoS), hospital costs, or less risk of developing chronic infection when treating an acute mesh infection.

The aim of the present study was to assess the short- and long-term outcomes after such a strategy (use of long-term absorbable mesh) for patients with infected ventral mesh requiring removal in an acute and chronic context.

## Methods

### Patients, design, and eligibility criteria

A retrospective cohort study was performed in two tertiary referral centers in France (Hautepierre Hospital, Strasbourg and Lyon Sud Hospital, Lyon). All consecutive patients who had a documented acute or chronic infection of a ventral mesh were screened. Patients who had their infected mesh explanted and insertion of a long-term absorbable mesh were included. Both elective and (semi-) emergency operations were included. Patients < 18 years old were excluded. The study period extended from January 1, 2017 to December 31, 2024.

### Outcomes and definitions

The primary outcome was the hernia recurrence rate. The assessment of hernia recurrence was based on clinical examination. If a recurrence was suspected and the patient was symptomatic, a CT-scan was performed. Secondary outcomes were 90-day postoperative complications, LoS, reoperation, need of removal of the long-term absorbable mesh, acute infection becoming chronic, and readmission rates. Postoperative complications were defined by the Clavien-Dindo classification [21]. Surgical-site occurrences comprised all wound related complications (including surgical-site infections) [22].

Infection of the primary mesh was suspected based on the clinical findings, radiological imaging (ultrasound, CT-scanner, magnetic resonance imaging), and laboratory results (leukocytes, C-reactive protein, procalcitonin, blood and periprosthetic fluid cultures). Clinical findings potentially related to mesh infection were wound discharge during several days (clear, purulent, hematic, or stercoral discharge), recurrent fever (defined as multiple episodes of temperature > 38° C), cellulitis around the scar (inflammatory erythema of the dermohypodermis around the surgical scar) or continuous pain on the mesh area. Radiological characteristics of mesh infection were presence of a fluid collection (density depending on the type of collection: hematoma, seroma, abscess) around the mesh, inflammation in the mesh area with inflammatory infiltration of surrounding tissue, presence of sinus tracts, mesh migration, crumpled mesh, or focal thickening of abdominal wall. The mesh was always sent for bacteriological analysis when removal and mesh infection was confirmed. Per definition, all included patients were grade 4 according to the Ventral Hernia Working Group (VHWG) classification [23] or grade 3c according to the modified VHWG classification [24]. Acute mesh infection was defined if infection occurred within 90 postoperative days, while chronic mesh infection was defined when mesh infection occurred after 90 postoperative days. Postoperative outcomes after acute and chronic infections were presented separately in the results.

### Management strategy

The management strategy was standardized and identical in both included centers. In acute infection, when it was decided to reoperate on the patient, complete removal of previous mesh was performed. In case of chronic infection, patients were included in a randomized controlled trial comparing primary herniorrhaphy to long-term absorbable mesh in contaminated fields [25]. Patients in the mesh group were included in this study. In the operation room (OR), potential remaining existing collections were drained and the mesh was removed in totality. At that time, the degree of contamination (presence of pus, stercoral contamination, non purulent infection) was evaluated. The surgeon then evaluated if a VAC therapy was needed or not. If a VAC therapy was put in place, patients were taken back to the OR within the next 72 h for removal of the VAC dressing and implantation of a new long-term absorbable mesh. If no VAC dressing was judged necessary during the first operation, a new long-term absorbable mesh was put in one stage procedure. One-stage operation was the routine and first-line procedure. Two-stage reconstruction was only considered in case of purulent or stercoral abscess or gross intraoperative surgical wound contamination. Patients with VAC therapy were taken back to the operation room 72 h after VAC implantation. If the wound was judged clean by the surgeon (i.e., absence of pus and undrained fluid collection), the new mesh was implanted, otherwise a new VAC dressing was applied for another planned look 3 days later. It was decided to routinely use a long-term absorbable poly-4-hydroxybutyrate mesh as the field was considered contaminated, and this mesh was deemed less at risk of getting infected and needing removal.

The long-term absorbable mesh that was used was a heavyweight (158 g/m^2^) poly-4-hydroxybutyrate mesh (Phasix©) with pore size of 0.35 mm. The size of the mesh was tailored to the hernia defect. This mesh has been shown to slowly resorb within 12–18 months in animal studies [26]. The mesh was preferentially placed in the retromuscular (sublay) space (first-line choice). If this space was not available or judged inadequate (prior dissection, infected space, destructed, resected or poor quality of posterior rectus sheath), the mesh was placed subcutaneously (onlay) or intraperitoneally. Choice between the use of intraperitoneal or subcutaneous spaces depended on several factors, such as location of previous infected mesh, intraoperative contamination, defect size, or quality of the abdominal wall.

When the mesh was placed intraperitoneally, the composite version of the poly-4-hydroxybutyrate mesh including hydrogel barrier (Phasix ST©) was used.

Patients with acute and chronic mesh infections did not routinely receive preoperative antibiotics, except in case of recurrent fever, high inflammatory syndrome, signs of severe infection (tachycardia/hypotension, neurological symptoms) or concomitant cellulitis. If antibiotics were required broad spectrum agents, such as piperacillin/tazobactam were prescribed. Prophylactic antibiotics using cefazolin before incision were given if patients were not already under antibiotherapy. Postoperatively, antibiotics were given for 7 to 14 days based on the results of the antibiogram of the wound, mesh, and blood cultures, intraoperative results, and discussion between the surgeon and the infectious disease specialist. Antibiotics were tailored to the type and sensitivity of the found bacteria during the postoperative period. Data on antibiotics and found bacteria were not available at time of data collection and not retrospectively collected in the database for the current study.

### Statistics

Descriptive statistics were used to summarize the cohort. Continuous variables were presented with median and interquartile range, while categorical data were summarized with number and percentage. Mean follow-up was calculated using the reverse Kaplan-Meier method. Range of follow-up (minimal and maximal values) was given in months.

SPSS 29.0 for Mac OS was used for all statistical analyses (IBM, Chicago, USA).

This study was approved by the internal review board of the Hospices Civils de Lyon (IRB 0013204, study number: 22_446).

## Results

A total of 29 patients were included in the study (13 from Strasbourg and 16 from Lyon): 14 acute infections and 15 chronic infections. Median age of the patient cohort was 71 years (IQR 54–73). Fifteen women and 14 men were included in the overall cohort. Median body mass index was 29 kg/m^2^ (IQR 27–35). Before mesh infection, 7 patients presented a primary hernia (umbilical or epigastric hernias) and 22 patients had an incisional hernia. Among the 22 patients with incisional hernia, 9 patients had undergone a urological surgery, 8 a gastrointestinal surgery, and 5 a gynecological procedure.

### Acute mesh infections (n = 14)

Details of the preoperative characteristics of patients with acute mesh infection are summarized in Table 1a. 29% of patients (4/14) were active smokers. Most of the patients had a midline hernia (11/14, 79%). Mesh locations were intraperitoneal in 9 patients (64%) and extraperitoneal in 5 patients (36%). A collection in contact with the mesh was present in half of the patients (*n* = 7). The median time between initial operation and mesh removal was 9 days (IQR 6–15).

**Table 1:** Preoperative characteristics of the included patients (*n* = 29)

	Median or number	IQR or percentage
a) Acute mesh infections (*n* = 14)		
Age, years	67	54–74
Sex (women/men)	6/8	43%/57%
BMI, kg/m^2^	28	26–34
Active smoking	4	29%
Diabetes mellitus	3	21%
ASA score I-II/III-IV	7/7	50%/50%
Immunosuppression	1	7%
COPD	1	7%
Cirrhosis	0	0%
History of cancer	3	21%
Locations of hernia Midline Lateral	11 3	79% 21%
Locations of previous mesh Intraperitoneal Extraperitoneal	9 5	64% 36%
Types of previous mesh Composite (polypropylene or polyester-based) Polypropylene only Poly-4-hydroxybutyrate	9 4 1	64% 29% 7%
Technique of previous surgery Open Minimally-invasive	6 8	43% 57%
Presentation of mesh infection Wound discharge Enteroprosthetic fistula Collection around mesh	5 2 7	36% 14% 50%
Time between initial operation and mesh removal, days	9	6–15
b) Chronic mesh infections (*n *= 15)		
Age, years	70	63–73
Sex (women/men)	9/6	60%/40%
BMI, kg/m^2^	29	27–36
Active smoking	2	13%
Diabetes mellitus	4	26%
ASA score I-II/III-IV	9/6	60%/40%
Immunosuppression	0	0%
COPD	3	20%
Cirrhosis	0	0%
History of cancer	6	40%
Locations of hernia Midline Lateral	12 3	80% 20%
Locations of previous mesh Intraperitoneal Onlay (subcutaneous)	13 2	87% 13%
Types of previous mesh Composite (polypropylene or polyester-based) Polypropylene only	13 2	87% 13%
Technique of previous surgery Open Minimally-invasive	3 12	20% 80%
Presentation of mesh infection Wound discharge Enteroprosthetic fistula Collection around mesh	9 2 4	60% 13% 27%
Time between initial operation and mesh removal, months	48	13–99
Botulinum toxin injection	2	13%

*BMI* body mass index, *ASA* American Society of Anesthesiologists, *COPD* chronic obstructive pulmonary disease, *IQR* interquartile range

Two patients had an initial loss of domain (Sabbagh index = hernia volume/peritoneal volume > 20%). All 14 operations were done by laparotomy. The median operation duration was 180 min (IQR 113–195). The median defect size after mesh removal was 8 cm (IQR 8–12). Most patients underwent a one-stage operation (*n* = 11, 79%) with infected mesh removal and implantation of a long-term absorbable mesh. Three patients (21%) had a VAC wound therapy in the context of a two-stage procedure. Posterior component separation was necessary in 4 patients to obtain closure of the posterior rectus sheath and good mesh overlap. In 86% of the patients (*n* = 12), the new mesh was placed in the retromuscular space. All intraoperative details are summarized in Table 2a.

**Table 2:** Hernia characteristics and intraoperative data of the included patients (*n* = 29).

	Median or number	IQR or percentage
a) Acute mesh infections (*n *= 14)		
Loss of domain	2	14%
Emergency surgery*	2	14%
Laparotomy	14	100%
Hernia defect size, cm	8	5–12
One-stage/two-stage procedure	11/3	79%/21%
Operation duration, min	180	113–195
Position of new mesh Retromuscular Intraperitoneal Onlay (subcutaneous)	12 2 0	86% 14% 0%
Posterior component separation	4	28%
b) Chronic mesh infections (*n* = 15)		
Loss of domain	1	7%
Emergency surgery*	1	7%
Laparotomy	14	100%
Hernia defect size, cm	7	3–10
One-stage/two-stage procedure	12/3	80%/20%
Operation duration, min	150	135–225
Position of new mesh Retromuscular Intraperitoneal Onlay (subcutaneous)	13 1 1	86% 7% 7%
Posterior component separation	3	21%

*Not planned as an elective operation

Nine patients (64%) developed postoperative complications after removal of infected mesh and reimplantation of a long-term absorbable mesh (within the first 90 postoperative days). Minor complication rate was 50% (7/14), while major complications occurred in 14% of the patients (2/14, one reoperation for drainage of a superficial surgical-site infection and one reoperation for drainage of an abdominal wall hematoma). Surgical-site occurrences, surgical-site infections, and infectious complications (excluding surgical-site infections) occurred in 11 (79%, 2 occurred after the two-stage procedure), 6 (43%, 1 occurred after the two-stage procedure), and 1 (7%) cases, respectively. The median LoS was 10 days (IQR 5–15). In total, 8 patients (54%) had to be reoperated on (5 within 90 days and 3 after 90 days). Within a mean follow-up of 17 months (95% CI 8–25), 3 patients (21%) presented a hernia recurrence. The new long-term absorbable mesh had to be explanted in 1 patient (7%) due to infectious contamination with clinical repercussions. No chronic infection developed with this management strategy. Short- and long-term outcomes of the 14 included patients are shown in detail in Table 3a.

**Table 3:** Postoperative outcomes of the included patients (*n* = 29)

	Median or number	IQR or percentage
a) Acute mesh infections (*n *= 14)		
Overall morbidity Minor complications Major complications	9 7 2	64% 50% 14%
Surgical-site occurrences	11	79%
Surgical-site infections	6	43%
Infectious complications*	1	7%
Length of stay, days	10	5–15
Reoperation within 90 days	5	36%
Reoperation after 90 days°	3	21%
Percutaneous drainage within 90 days	1	7%
Percutaneous drainage after 90 days°	0	0%
Recurrence of hernia°	3	21%
New mesh removal°	1	7%
b) Chronic mesh infections (*n*=15)		
Overall morbidity Minor complications Major complications	9 7 2	60% 47% 13%
Surgical-site occurrences	5	33%
Surgical-site infections	2	13%
Infectious complications*	1	7%
Length of stay, days	7	5–13
Reoperation within 90 days	1	7%
Reoperation after 90 days°	0	0%
Percutaneous drainage within 90 days	2	13%
Percutaneous drainage after 90 days°	0	0%
Recurrence of hernia°	0	0%
New mesh removal°	0	0%

* Excluding surgical-site infections

° During a mean follow-up of 17 months (95% CI 8-25). Range of follow-up was 3 months (minimum) and 77 months (maximum)

### Chronic mesh infections (n= 15)

Characteristics of patients with chronic mesh infection are shown in Table 1b. Twelve patients had a recurrent midline hernia (12/15, 80%), while 3 patients had a recurrent lateral hernia (20%). Most patients had an infected mesh located intraperitoneally (14/15, 93%). The most common clinical presentation was chronic wound discharge (9/15, 60%). The median time between mesh implantation and mesh removal was 48 months (IQR 13–99).

All reoperations for mesh removal were performed by laparotomy. Median defect size once the mesh was removed was 7 cm (IQR 3–10). Three patients underwent a two-stage procedure with first infected mesh removal and subcutaneous VAC wound therapy, followed by implantation of a new mesh and skin closure in the subsequent days (20%). In 13 patients (87%), the new long-term absorbable mesh was positioned in the retromuscular space. Intraoperative details can be found in Table 2b.

Overall postoperative morbidity was 60% (9/15), with minor complications occurring in 47% (7/15). Median LoS was 7 days (IQR 5–13). Only one patient had to be reoperated on during the first 90 postoperative days. No hernia recurrence and no mesh removal occurred within a mean follow-up of 17 months (95% CI 8–25). Postoperative outcomes are depicted in Table 3b.

### One-stage vs. two-stage procedure

Overall, 6 patients underwent a two-stage procedure and 23 patients a one-stage procedure. Same complication and recurrence rates were found between patients in the one- and two-stage groups (complications: 19/23 vs. 3/6, *p* = 0.096 and recurrence: 3/23 vs. 0/6, *p* = 0.350). No difference of length of hospital stay was also found between both groups (median: 5 days, IQR 3–41 vs. 10 days, IQR 4–15, *p* = 0.573).

## Discussion

This study assessing the use of a long-term absorbable mesh after removal of an infected mesh in acute and chronic conditions showed that postoperative complications occurred frequently but that the incidence rates of new mesh explantation and hernia recurrence were low.

The current study evaluated the outcomes of the use of a long-term absorbable poly-4-hydroxybutyrate mesh in contaminated fields (i.e., in case of previous infected mesh requiring removal). Some studies have suggested that long-term absorbable meshes such as poly-4-hydroxybutyrate mesh have theoretically better resistance in infected fields, making them ideal in case of removal of an infected synthetic mesh [18]. The theoretical and mechanistic advantages of using such a mesh are multiple. First, poly-4-hydroxybutyrate stimulates the expression of anti-microbial peptides in macrophages, therefore decreasing the risk of bacterial colonization and of surgical-site infection [27]. It should be noted that this property was found in vitro [27]. It is currently unclear if the same effect is found in vivo and if a contaminated field influences the biological response, which could explain the relatively high rate of surgical-site infections found after acute mesh infection (43%). Moreover, the monofilament biosynthetic scaffold with open pores facilitates rapid tissue incorporation, rendering the mesh potentially less sensitive to bacterial infection and better penetration of antibiotics in case of bacterial contamination. It has also been postulated to that lower immunogenicity induced by an increase anti-inflammatory response promoted by the poly-4-hydroxybutyrate compared to non absorbable synthetic meshes could play a positive role in an infectious context. In addition, as the mesh is resorbable (via hydrolysis occurring at a predictable and relatively stable rate), the risk of mesh infection occurring at a late stage could be decreased. Another possible advantage would be that if a hernia recurs as the infection increases the risk of recurrence, treatment options and specific planes are not compromised because the mesh would have been absorbed. Bueno-Lledo et al. published in 2021 a series of 30 patients with chronic mesh infection who underwent mesh removal and simultaneous implantation of poly-4-hydroxybutyrate mesh (single-stage management) [19]. They found a low hernia recurrence rate with only 3.3% of patients who presented a hernia recurrence within a median follow-up of 34.5 months. Of note, inguinal mesh infections were also included (*n* = 4), which was not the case in the present study focusing on ventral hernia. No hernia recurrence within 17-month follow-up was found in this study after chronic infection. In acute infection, 21% of patients presented a hernia recurrence, which remains within the range found in the literature for poly-4-hydroxybutyrate use in complex hernia repairs or in contaminated fields [28, 29]. Literature on recurrence rate after acute mesh infection remains scarce, as most data arise from chronic mesh infection, and most studies do not separate acute and chronic infections. In a retrospective review of the data from the Veterans Health Administration on mesh removal for infection within 5 years (no precisions on timing), Dipp Ramos et al. found that all patients who had only mesh removal without hernia repair developed a recurrence, while 49% of patients after subsequent hernia repair with mesh (biological mesh: 107/166 = 65%, synthetic mesh: 59/166 = 35%) presented a recurrence [3]. A systematic review and single-center study published in 2018 by Shubinets et al. showed that the recurrence rate in 43 patients with mesh explant for infection (no precision on timing) was 67% (2/3) if a two-stage procedure (reconstruction with mesh after VAC therapy) was performed and 35% (14/40) if a single-stage procedure (reconstruction with mesh at the same time as mesh removal) was done [30]. In the two-stage procedure (*n* = 3), 2 biological (Permacol© and SurgiMend©) and 1 synthetic (Ventralight ST©) meshes were used, while in the single-stage procedure (*n* = 40) 34 biological (15 XenMatrix©, 14 Permacol©, 4 SurgiMend©, and 1 Strattice©) and 1 synthetic (Vicryl©) meshes were used (5 unknown). In a retrospective cohort of patients (*n* = 47) with median time to infection of 27 months, Alimi et al. found that mesh removal with VAC therapy followed by hernia repair with biological mesh (Strattice©) yielded a 11% recurrence rate, but the mean follow-up was only 10 months [31]. It nevertheless remains unclear if a long-lasting repair with similar results as repair with synthetic mesh can be achieved with this strategy.

Postoperative complications within 90 days occurred in 64% (acute infection) and 60% (chronic infection) of the patients of the cohort, encompassing mainly minor complications and surgical-site occurrences. This high morbidity reflects the septic situation leading to increased perioperative risk and the risk of wound-related events. Moreover, patients with acute infection or chronic sepsis often are multimorbid and have a persisting inflammatory state, increasing the risk of complications. Prior series of patients undergoing mesh removal showed morbidity rates around 40–70% [19, 28, 29]. In the present studied cohort, all deviations from the ideal postoperative course even minor, such as electrolyte imbalance, asymptomatic seroma or transient urinary retention, were considered and classified as postoperative complications, which can induce some differences when comparing to other studies.

The benefits of the presented strategy that were found in the current study were the absence of chronic infection development in patients who were treated for an acute infection and a relatively low median LoS (between 7 and 10 days). This could potentially lead to cost reduction compared to the strategy of aiming to save the infected mesh, but no control group was included in this study and financial data were not available. Only 1 long-term absorbable mesh had to be explanted and was removed at the beginning of the implementation of the presented strategy. In their article including 30 patients, Bueno-Lledo et al. showed that no patient had to have their poly-4-hydroxybutyrate mesh removed with their one-stage strategy (mesh removal and simultaneous implantation of a long-term absorbable mesh) [19]. In a meta-analysis on ventral hernia repair in high-risk patients and contaminated fields, Morales-Conde et al. found that the pooled rate of synthetic mesh removal was 9% [29]. In this study, patients with modified VHWG classification grade 2 (clean-contaminated) and 3 (contaminated) were included. This represents an important heterogeneity between the studies to consider, whereas in the present study only patients with modified VHWG classification grade 3c were included (infected mesh). There are currently few data specifically assessing the outcomes of ventral hernia mesh repair in case of infected field, corresponding to grade 3c of the modified VHWG classification. The presented strategy also had disadvantages that need to be acknowledged. First, it can be seen as excessive in case where infected mesh could have been saved. Moreover, long-term data on the solidity of the repair and hernia recurrence risk remain unknown. The goal of this study was to present the outcomes of such a strategy and to show that it could be a management option in case of mesh infection. The presented strategy could be a complementary option to mesh salvage, interesting to have in our management armamentarium, especially in patients with high risk of mesh salvage impossibility (i.e., polytetrafluoroethylene mesh, intraperitoneal mesh, or enteroprosthetic fistula).

The present study has some limitations that need to be mentioned. First, this was a retrospective study with data retrieved from patient charts. This can induce some mistakes and a collection bias. Moreover, there were some missing data. Secondly, the cohort is relatively small with 29 patients included. However, the incidence of patients requiring prior mesh removal for infection is low even in referral, specialized, and tertiary centers. In addition, as only patients with infected mesh (grade 3c of the modified VHWG classification) and no other type of contaminations (clean-contaminated or 3a/3b grades) were included, the homogeneity of the cohort was improved facilitating data interpretation and future comparisons. Another limitation was the absence of a control group or comparator (e.g., biological mesh, suture repair, or mesh salvage). This decreases the strength of the conclusion that can be drawn from this article. It must be kept in mind that depending on individual case conditions mesh salvage can be achieved with antibiotherapy and surgical wound debridement in a high number of cases. The presented strategy can be used in specific situations where multiple risk factors of conservative treatment failure are present. It can also be mentioned that the median follow-up (17 months) was potentially not long enough to find recurrence as the resorption time of the poly-4-hydroxybutyrate is estimated to be between 12 and 18 months. The fact that the decision to perform a one- or two-stage procedure was left to the surgeon’s discretion might limit the reproducibility of the presented strategy. Finally, data on antibiotherapy prescription and duration, and types of micro-organisms were not available at time of data collection and were not included in the database of this study.

Based on the current findings, future studies should strive to specify predictive criteria defining when the presented strategy gives the best outcomes in case of infected mesh. Moreover, the antibiotherapy strategy and location of the mesh placement in this context remain unelucidated.

In conclusion, mesh removal followed by implantation of a long-term absorbable mesh with or without prior VAC therapy had in this cohort a high postoperative morbidity but good long-term results in terms of mesh removal and hernia recurrence rate. Prospective data and larger cohorts would help to confirm the preliminary outcomes of the use of long-term absorbable meshes in patients with prior infected mesh in acute and chronic contexts.
